# HER2 Status in RAS and BRAF Wild-Type Metastatic Colorectal Cancer: A Portuguese Study

**DOI:** 10.7759/cureus.42536

**Published:** 2023-07-27

**Authors:** Teresa Fraga, Maria João de Sousa, Joana Magalhães, Raquel Basto, Judy Paulo, Nuno Bonito, José Paulo Magalhães, Paulo Figueiredo, Gabriela M Sousa

**Affiliations:** 1 Medical Oncology, Instituto Português de Oncologia de Coimbra Francisco Gentil, Coimbra, PRT; 2 Medical Oncology, Centro Hospitalar Universitário do Porto, Porto, PRT; 3 Medical Oncology, Centro Hospitalar Vila Nova de Gaia/Espinho, Gaia, PRT; 4 Pathology, Instituto Português de Oncologia de Coimbra Francisco Gentil, Coimbra, PRT

**Keywords:** braf mutation, portugal, ras, metastatic, colorectal cancer, her2

## Abstract

Introduction: Colorectal cancer (CRC) is the second-most deadly cancer worldwide. However, there remains a scarcity of precision treatments available for this type of cancer. Amplification or overexpression of human epidermal growth factor receptor 2 (HER2+) is a well-established therapeutic target in gastric and breast cancer. HER2 is positive in approximately 5% of CRC cases and has been implicated in resistance to therapy with anti-epidermal growth factor receptor antibodies. The aim of this study was to evaluate HER2 status in RAS and BRAF wild-type metastatic CRC (mCRC) and its correlation with survival outcomes.

Materials and methods: A single-center retrospective analysis of RAS and BRAF wild-type mCRC patients undergoing systemic treatment was conducted from July 2014 to September 2020. Tissue HER2 status was determined by immunohistochemistry (IHC) and/or ﬂuorescence in situ hybridization (FISH) and/or chromogenic in situ hybridization (CISH). HER2+ was deﬁned as IHC3 (+) or IHC2 (+) through FISH or CISH (+).

Results: Fifty-nine patients were included. The median age of all the included patients was 64 years (33-82). Four patients had HER2+ tumors (7%). Four patients had HER2+ tumors (7%). The majority of HER2+ mCRC cases were males (n=3) and left-sided CRC (n=3). All patients received FOLFIRI plus cetuximab as ﬁrst-line treatment. At the median follow-up of 24.0 months, patients with HER2-negative mCRC presented with a median overall survival (mOS) of 39.4 months (95% conﬁdence interval (CI) 32.7-46.0) and the four patients with HER2+ mCRC had a mOS of 20.4 months (95% CI; 9.5-31.3; p=0.07). In HER2-negative patients, the median PFS (mPFS) was 11.3 months (95% CI; 9.2-13.4) vsHER2-positive patients with a mPFS of 10.9 months (95% CI; 1.3-20.4; p=0.47).

Conclusions: To our knowledge, this is the ﬁrst study reporting HER2+ in mCRC patients in a Portuguese population and the HER2+ rate was consistent with previous studies. Our study suggests that HER2+ may potentially be a marker that is able to predict poor prognosis in RAS and BRAF wild-type mCRC.

## Introduction

According to GLOBOCAN 2020, colorectal cancer (CRC) is currently the third-most commonly diagnosed cancer and the second-leading cause of cancer death in the world [1]. With an increase in screening lately, there has been a decrease in the trend of late-stage diagnoses; however, 25-30% of CRC patients still continue to present with metastatic disease, and about 10 to 30% of early-stage patients will develop a recurrence after curative surgery [2-6].

Over the last 20 years, significant efforts have been made to improve the treatment of metastatic CRC (mCRC) resulting in the improvement of the median overall survival (mOS) from about 10 to 30 months [7-11]. This progress was in part due to the development of new biological agents given in combination with chemotherapy as well as successful surgical treatment options [12]. However, their effectiveness is limited, with a five-year survival rate for mCRC of 10%, creating the need for novel treatment strategies [2,13].

The human epidermal growth factor receptor 2 (HER2) is a member of the epidermal growth factor receptor (EGFR) family of tyrosine kinase receptors and plays a crucial role in cell proliferation and differentiation, inhibition of apoptosis and tumor progression [13-16]. HER2 is a well-known therapeutic target in patients with breast and gastric cancer in which overexpression or amplification of HER2 (HER2-positive) is reported in 13-20% and 7-34% of cases, respectively [14]. In CRC, overexpression or amplification of HER2 has been described in 2% to 9.5% of patients; KAS wild-type CRC is more prevalent and occurs more frequently on the left side [10,11,14,15,17-19]. Although some research has reported HER2 status in mCRC patients as a mechanism of treatment resistance, this association has not been fully elucidated [5,10,13,20].

In this context, clinical trials explored the role of anti-HER2 treatment in HER2+ mCRC. Various anti-HER2 agents were tested in phase II trials from trastuzumab plus lapatinib (HERACLES-A trial), trastuzumab plus pertuzumab (MyPathway trial), and trastuzumab plus tucatinib (MOUNTAINEER trial), with objective response rates (ORR) of 30%, 32% and 55% and median progression-free survival (mPFS) of 4.7, 2.9 and 6.2 months, respectively [10,13,16,18,21-23]. More recently, the phase II trial DESTINY-CRC01 of trastuzumab deruxtecan revealed an ORR of 45%, showing benefit even in mCRC patients with prior anti-HER2 treatments (ORR of 43.8%) [16,21,24]. On the contrary, the combination of pertuzumab and trastuzumab-emtansine, evaluated in a phase II trial (HERACLES-B trial) did not reach its primary endpoint of ORR [21,25].

Our research did not find studies that assessed the incidence of HER2+ in Portuguese patients with mCRC. As such, in this study, we aimed to evaluate the HER2 status in mCRC patients in a Portuguese cancer center and its correlation with survival outcomes.

This article was previously presented as a poster at the 2021 Molecular Analysis for Precision Oncology on October 7-9, 2021.

## Materials and methods

Study design

This was an observational, retrospective, and single-center study approved by the institutional ethics committee and conducted in accordance with the principles of the Declaration of Helsinki and the International Conference on Harmonization Good Clinical Practice guidelines. Patients with RAS and BRAF wild-type mCRC that underwent systemic treatment from July 2014 to September 2020 were analyzed.

Sample selection

The determination of HER2-positive was based on the pathology determination of all patients diagnosed with mCRC that met the inclusion criteria. The inclusion criteria were: ≥18 years of age, histological confirmation of colorectal cancer with RAS (KRAS or NRAS) and BRAF wild-type status, treatment with at least one palliative chemotherapy regimen including fluoropyrimidine-, oxaliplatin- or irinotecan-based chemotherapy, anti-VEGF or anti-EGFR agents. Patients with oligometastatic disease treated with curative intent, patients without enough tumor tissue for immunohistochemical evaluation, and patients with incomplete or inaccurate clinical registries were excluded.

Clinical and pathological data

This study reviewed the medical records of the included patients. Data regarding the age, sex, Eastern Cooperative Oncology Group Performance Status (ECOG-PS), HER2, BRAF, mismatch repair deficiency (dMMR) status, primary tumor location (right or left-sided colon cancers according to embryologic origin), location of metastasis, clinical and pathological staging (American Joint Committee on Cancer (AJCC) 8th edition), first line of chemotherapy and tumor response (Response Evaluation Criteria in Solid Tumors (RECIST) 1.1) was collected.

Molecular data

Tumor tissue HER2 status was determined by performing immunohistochemistry (IHC) and/or ﬂuorescence in situ hybridization (FISH) and/or chromogenic in situ hybridization (CISH) as defined by the HERACLES diagnostic criteria. HER2+ was deﬁned as either IHC (3+) or IHC (2+) through FISH or CISH (+). A Ventana BenchMark ULTRA (Roche Diagnostics, Basel, Switzerland) with anti-HER-2/neu (4B5) rabbit monoclonal primary was used for the assays.

Study assessments and outcome measures

The primary endpoint of the study was to evaluate the incidence of HER2-positive in patients with mCRC RAS and BRAF wild-type. The secondary objectives were (1) demographic and clinicopathological characterization of the patients according to HER2 status and (2) OS and PFS according to patients' HER2 status. OS was calculated from the time of mCRC diagnosis to death or date of last follow-up and PFS was determined from the time of treatment initiation until disease progression, death, or date of last follow-up.

Statistical analysis

Demographic characteristics and treatment details were retrieved from medical records. Categorical variables related to demographic and baseline characteristics were summarized using frequencies and percentages. PFS and OS were analyzed using the Kaplan-Meier method and further characterized in terms of the median with the two-sided 95% confidence intervals (CI).

## Results

Study population - clinicopathological features

A total of 59 patients were included in this study. There were four HER2-positive cases (7%). A summary of the baseline and clinical characteristics of the patients is presented in Table 1. The median age of the sample was 64 years (33-82), with 29 men (49.2%) and 30 women (50.8%). A total of 45 patients (76.3%) had an ECOG-PS of 0 and 14 (23.7%) had an ECOG-PS of 1. More than half of the patients had metastatic disease at presentation (n=33, 55.9%). The majority of patients had left-sided tumors (n=50, 84.7%) and the most common site for metastasis was the liver (n=36, 61.0%) followed by the lung (n=24, 40.7%) and peritoneal carcinomatosis (n=12, 20.3%). The most frequent first-line regime of chemotherapy was FOLFIRI plus cetuximab (n=51, 86.4%).

**Table 1:** Baseline patient demographics and clinical characteristics ECOG-PS: Eastern Cooperative Oncology Group - Performance Status; FOLFIRI: Folinic acid, fluorouracil and irinotecan; FOLFOX: Folinic acid, fluorouracil and oxaliplatin; max: maximum; min: minimum; n:* *number of patients; %: percentage

Variable	n	%
Patient age		
< 65 years	31	52.5
≥ 65 years	28	47.5
Gender		
Male	29	49.2
Female	30	50.8
ECOG-PS		
0	45	76.3
1	14	23.7
Stage at initial diagnosis		
I	1	1.7
II	5	8.5
III	20	33.9
IV	33	55.9
Tumor location		
Right-sided	9	15.3
Left-sided	50	84.7
Number of metastatic sites		
≤2	53	89.8
>2	6	10.2
Metastatic sites		
Liver	36	61
Lung	24	40.7
Peritoneal carcinomatosis	12	20.3
Other location	13	22
First-line treatment		
FOLFIRI+cetuximab	51	86.4
Other	8	13.6
FOLFIRI	4	6.8
FOLFIRI+panitumumab	1	1.7
FOLFOX	1	1.7
DeGramont	1	1.7
Cetuximab monotherapy	1	1.7

HER2 status and clinicopathological features

The clinical and pathological features according to the patient's HER2 status are presented in Table 2. The median age of HER2-positive cases was similar to that of negative cases (65 vs 64 years). In the HER2-negative subgroup, the majority of patients were female (n=29, 52.7%), mostly with an ECOG-PS of 0 (n=42, 76.4%) and left-sided tumors (n=47, 85.5%). Furthermore, more than half of the patients had one or two metastatic sites (n=50, 90.9%), with the most common being the liver (n=32, 58.2%), followed by the lung (n=21, 38.2%). In the HER2-positive subgroup, three (75%) were male, three (75%) had an ECOG-PS of 0, and three (75%) had left-sided mCRC. All four of the HER2+ patients had liver metastases, while three of them had lung metastases as well. The majority of the HER2-negative patients and the entire cohort of HER2-positive patients received FOLFIRI plus cetuximab as ﬁrst-line treatment.

**Table 2:** HER2-negative and HER2-positive population characteristics ECOG-PS: Eastern Cooperative Oncology Group - Performance Status; FOLFIRI: folinic acid, fluorouracil and irinotecan; FOLFOX: folinic acid, fluorouracil and oxaliplatin; HER2: human epidermal growth factor receptor 2; max: maximum; min: minimum; n: number of patients; %: percentage

	HER2- (*n*=55)		HER2+ (*n*=4)	
Median age, years (min-max)	64 (33-82)		65 (54-72)	
Variable	n	%	n	%
Patient age				
< 65 years	29	52.7	2	50
≥ 65 years	26	47.3	2	50
Gender				
Male	26	47.3	3	75
Female	29	52.7	1	25
ECOG-PS				
0	42	76.4	3	75
1	13	23.6	1	25
Stage at initial diagnosis				
M0	26	47.3	-	-
M1	29	52.7	4	100
Tumor Location				
Right-sided	8	14.5	1	25
Left-sided	47	85.5	3	75
Number of metastatic sites				
≤2	50	90.9	3	75
>2	5	9.1	1	25
Metastatic sites				
Liver	32	58.2	4	100
Lung	21	38.2	3	75
Peritoneal carcinomatosis	10	18.2	2	50
Other Location	13	23.6	4	100
First-line treatment				
FOLFIRI+cetuximab	47	85.5	4	100
Other	8	14.5	-	-
FOLFIRI	4	50	-	-
FOLFIRI+panitumumab	1	12.5	-	-
FOLFOX	1	12.5	-	-
DeGramont	1	12.5	-	-
Cetuximab monotherapy	1	12.5	-	-

The effect of HER2 status on relative survival

**Figure 1:**
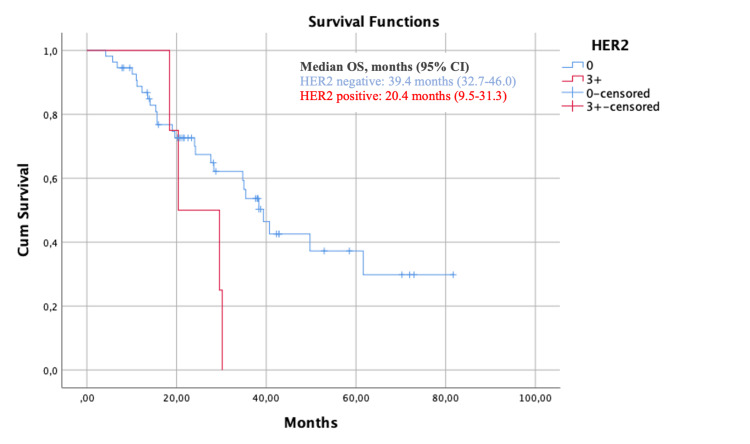
Kaplan–Meier Estimates of overall survival (OS) in the HER2-negative population HER2: human epidermal growth factor receptor 2; OS: overall survival

**Figure 2:**
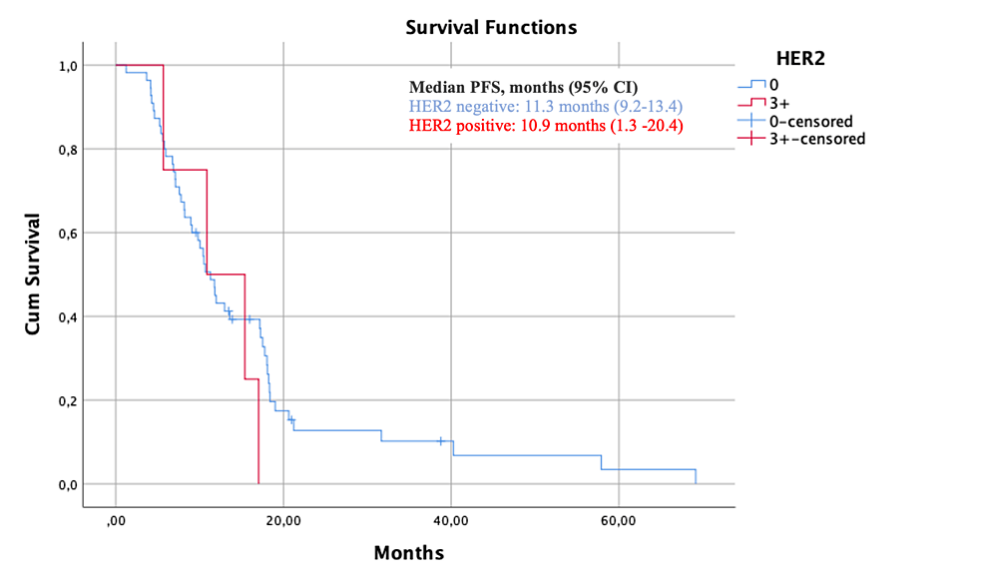
Kaplan–Meier estimates of progression-free survival (PFS) in the HER2-negative population HER2: human epidermal growth factor receptor 2; PFS: progression-free survival

At a median follow-up of 24.0 months, the median OS of the HER2 negative cohort was 39.4 months (95% CI 32.7-46.0) and the median OS of the HER2+ cohort was 20.4 months (95% CI 9.5-31.3) (p=0.07) (Figure 1). As for PFS, the median PFS of the HER2-negative cohort was 11.3 months (95% CI 9.2-13.4) and the median PFS of the HER2+ cohort was 10.9 months (95% CI 1.3-20.4) (p=0.47) (Figure 2). 

## Discussion

HER2 overexpression or amplification may define a new subset of patients with a potential target in mCRC. The overexpression rate of HER2 in our study was 7%, comparable to the incidence reported by Sawada et al at 7.7%, Sartore-Bianchi et al.* *at 6.7%, and Valtorta et al.* *and Richman et al.at approximately 5% [20,26-28].

Nearly a quarter of patients with CCR present with metastases at the time of diagnosis [2,29]. More than half of our patients had metastatic disease at presentation (n=33, 55.9%) and all four of the HER2+ patients had metastasis at diagnosis, with no statistical difference (p=0.34). This is similar to the population described by Sartore-Bianchi et al. and Sawada et al., where the majority of HER2+ patients were stage IV at diagnosis [20,28]. 

In the HER2+ cohort from our study, all the patients had liver metastasis and three had lung metastasis. This high prevalence of liver and lung metastasis in this subgroup of patients, although with no statistical significance (lung metastasis, p=0.29; liver metastasis, p=0.15), is similar to the results from Sawada et al. [28]. 

In previous studies, HER2 amplification was highly prevalent in left-sided CRC with reports showing that HER2-positive CRCs are mutually exclusive with KRAS, NRAS, and BRAF mutations [20,29,30]. This is also present in our results, where three patients (n=3/4) with HER2-positive mCRC had the primary tumor on the left side, but with no statistical significance (p=0.49). Consequently, patients with these characteristics could be good candidates for further HER2 expression examination.

Combined data from the QUASAR, FOCUS, and PICCOLO studies (n=3265; 1342 patients in stage IV) saw no association between HER2 expression and survival outcomes, in both OS and PFS [15]. Contrary to these findings, studies by Osako et al. and Kapitanović et al. HER2 overexpression was reported as an independent and negative prognostic indicator of CRC [15]. Furthermore, in the retrospective analysis done by Heppner et al. (1645 patients, stages I to IV CRC) was reported poorer OS in patients with HER2+ when compared to those without amplification, with the same results coming from the PETACC-8 study (3.8% patients with HER2+) that showed significantly lesser time to recurrence (HR 1.55; 95% CI 1.02-2.36; p=0.04) and shorter OS (HR 1.57; 95% CI 0.99-2.5; p=0.04) [15]. In our study, the results were similar to the studies that reported a worse outcome for patients with HER2+, with a tendency towards less OS and PFS.

Considering study limitations, the retrospective study design and small sample size which might have limited the interpretation of HER2-positive analyses. Despite these limitations, real-world data is extremely important to consolidate clinical trials’ results. 

## Conclusions

While HER2 amplification or overexpression is only present in a small percentage of mCRC, evidence continues to grow in support of HER2 status assessment in these patients. There is potential that with the continued evolution of data in this area, HER2 may become a validated therapeutic target. To our knowledge, this is the first study reporting the incidence of HER2 in mCRC patients in a Portuguese population and the HER2+ rate was consistent with previous studies. Our study suggests that HER2+ may potentially be a marker that is able to predict poor prognosis in RAS and BRAF wild-type mCRC.
